# RND Efflux Systems Contribute to Resistance and Virulence of *Aliarcobacter butzleri*

**DOI:** 10.3390/antibiotics10070823

**Published:** 2021-07-06

**Authors:** Cristiana Mateus, Ana Rita Nunes, Mónica Oleastro, Fernanda Domingues, Susana Ferreira

**Affiliations:** 1 CICS-UBI-Health Sciences Research Centre, University of Beira Interior, 6200-506 Covilhã, Portugal; cristiana.lopes.mateus@ubi.pt (C.M.); anaritalmeidan@hotmail.com (A.R.N.); fcd@ubi.pt (F.D.); 2 National Reference Laboratory for Gastrointestinal Infections, Department of Infectious Diseases, National Institute of Health Dr. Ricardo Jorge, Av. Padre Cruz, 1649-016 Lisbon, Portugal; monica.oleastro@insa.min-saude.pt

**Keywords:** *Aliarcobacter butzleri*, RND efflux pumps, virulence, resistance

## Abstract

*Aliarcobacter butzleri* is an emergent enteropathogen that can be found in a range of environments. This bacterium presents a vast repertoire of efflux pumps, such as the ones belonging to the resistance nodulation cell division family, which may be associated with bacterial resistance, as well as virulence. Thus, this work aimed to evaluate the contribution of three RND efflux systems, AreABC, AreDEF and AreGHI, in the resistance and virulence of *A. butzleri*. Mutant strains were constructed by inactivation of the gene that encodes the inner membrane protein of these systems. The bacterial resistance profile of parental and mutant strains to several antimicrobials was assessed, as was the intracellular accumulation of the ethidium bromide dye. Regarding bacterial virulence, the role of these three efflux pumps on growth, strain fitness, motility, biofilm formation ability, survival in adverse conditions (oxidative stress and bile salts) and human serum and *in vitro* adhesion and invasion to Caco-2 cells was evaluated. We observed that the mutants from the three efflux pumps were more susceptible to several classes of antimicrobials than the parental strain and presented an increase in the accumulation of ethidium bromide, indicating a potential role of the efflux pumps in the extrusion of antimicrobials. The mutant strains had no bacterial growth defects; nonetheless, they presented a reduction in relative fitness. For the three mutants, an increase in the susceptibility to oxidative stress was observed, while only the mutant for AreGHI efflux pump showed a relevant role in bile stress survival. All the mutant strains showed an impairment in biofilm formation ability, were more susceptible to human serum and were less adherent to intestinal epithelial cells. Overall, the results support the contribution of the efflux pumps AreABC, AreDEF and AreGHI of *A. butzleri* to antimicrobial resistance, as well as to bacterial virulence.

## 1. Introduction

*Aliarcobacter* genus belongs to the *Arcobacteraceae* family, with five other genera, *Arcobacter*, *Pseudarcobacter* gen. nov., *Halarcobacter* gen. nov., *Malaciobacter* gen. nov. and *Poseidonibacter* gen. nov., and a candidate genus, *Arcomarinus* [1,2,3]. *Aliarcobacter* genus includes 11 species, of which 8 have been validly published, *A. butzleri*, *A. cibarius*, *A. cryaerophilus*, *A. faecis*, *A. lanthieri*, *A. skirrowii*, *A. thereius* and *A. trophiarum* [2]. Bacteria from this genus are Gram-negative, small, curved rods and present motility by a single polar flagellum [1]. They can be found in a wide range of hosts and environments and isolated from different sources [4,5,6]. Among these species, *A. butzleri*, *A. cryaerophilus*, *A. skirrowii*, *A. thereius* and *A. lanthieri* have been associated with human disease, such as bacteremia, enteritis, diarrhea, abdominal cramps, nausea and vomiting [7,8,9,10]. In addition, *A. butzleri* and *A. cryaerophilus* have been recognized by the International Commission on Microbiological Specifications for Food as a serious hazard to human health [11]. When considering *A. butzleri*, several virulence mechanisms have been described for this species, such as adherence, invasion and cytotoxic effects [5,6,7]. Diseases caused by *A. butzleri* are usually self-limiting; nonetheless, several reports have described the use of antibiotics for its treatment, mostly with β-lactams, fluoroquinolones and macrolides [5]. However, *A. butzleri* has shown resistance to major classes of antibiotics, such as fluoroquinolones, macrolides, β-lactams, aminoglycosides and tetracyclines, with a considerable portion of strains being classified as multidrug-resistant [5,7]. Therefore, this bacterium has attracted attention due to its wide distribution and antibiotic resistance, even if the underlying mechanisms are still poorly understood. The only mechanism that has been linked to *A. butzleri* antibiotic resistance is a target modification, associated with resistance to fluoroquinolones [12]. More recently, the role of an efflux pump in the erythromycin-resistance phenotype was evidenced [13,14].

Efflux pumps are relevant elements for antimicrobial resistance, contributing to antibiotic resistance and also to bacterial virulence [15,16]. These systems comprise membrane proteins that are involved in the efflux of antibiotics and other toxic substances from the cell, being classified into six families: ATP-binding cassette (ABC), multidrug and toxic compound extrusion (MATE), major facilitator superfamily (MFS), proteobacterial antimicrobial compound efflux (PACE), resistance–nodulation–division (RND) and small multidrug resistance (SMR) [17]. Among these families, the superfamily of RND efflux pumps is the most relevant among multidrug resistance efflux transporters since, when overexpressed, bacteria may exhibit significant levels of resistance [18]. In addition, these systems may have a broad substrate specificity, due to their ability to recognize substrates of different sizes and with diverse properties [19]. The RND family of efflux pumps are tripartite protein complexes found in Gram-negative bacteria, which include an inner membrane protein, an outer membrane protein, and a periplasmic membrane fusion protein [16,19]. Efflux pumps from this family, such as CmeABC of *Campylobacter jejuni*; MexAB–OprM of *Pseudomonas aeruginosa*; or AcrAB of *Enterobacter cloacae*, *Klebsiella pneumoniae*, *Salmonella enterica* and others, were shown to play multiple functions, being involved in resistance to compounds with different structures, such as bile salts, various classes of antibiotics, detergents and dyes, and also contributing to the fitness, survival and virulence of these bacteria [16,20,21,22,23,24].

Genetic studies conducted by Isidro et al. (2020) have identified 19 putative efflux systems in the genome of *A. butzleri*, with 8 belonging to the efflux pumps of the RND family. Among these are the AreABC (EP16), AreDEF (EP15) and AreGHI (EP4) systems, for which complete operons were found at the genomic level [13]. Nonetheless, until now, only the AreABC system has been characterized, and it was shown to play a role in resistance to several antibiotics, particularly to erythromycin [14]. Increasing the knowledge regarding the role of efflux systems in antimicrobial resistance and virulence of the emerging pathogen *A. butzleri* is, therefore, a promising line of investigation. With this work, we aimed to elucidate the role that the AreABC, AreDEF and AreGHI RND efflux pumps play in *A. butzleri* antibiotic resistance and virulence.

## 2. Results and Discussion

### 2.1. Impact of the Inactivation of Three RND Efflux Pumps on Bacterial Resistance

Bacterial resistance to various antimicrobial agents has been linked to efflux pumps that are responsible for the extrusion of these agents [18]. RND efflux pumps stand out for their role in resistance to multiple antimicrobials in Gram-negative bacteria, due to their broad substrate specificity, including (1) antibiotics, such as penicillins, cephalosporins, fluoroquinolones, macrolides, chloramphenicol and tetracyclines; (2) cationic dyes, such as crystal violet and ethidium bromide; and (3) detergents such as Triton X-100, biocides, heavy metals, organic solvents and host-derived molecules. The extruded compounds may present diverse structure charges, among other characteristics; however, their lipophilicity, or the presence of lipophilic domains, has been described as a common feature among the substrates of these systems [15,16,19,25].

**Figure 1:**
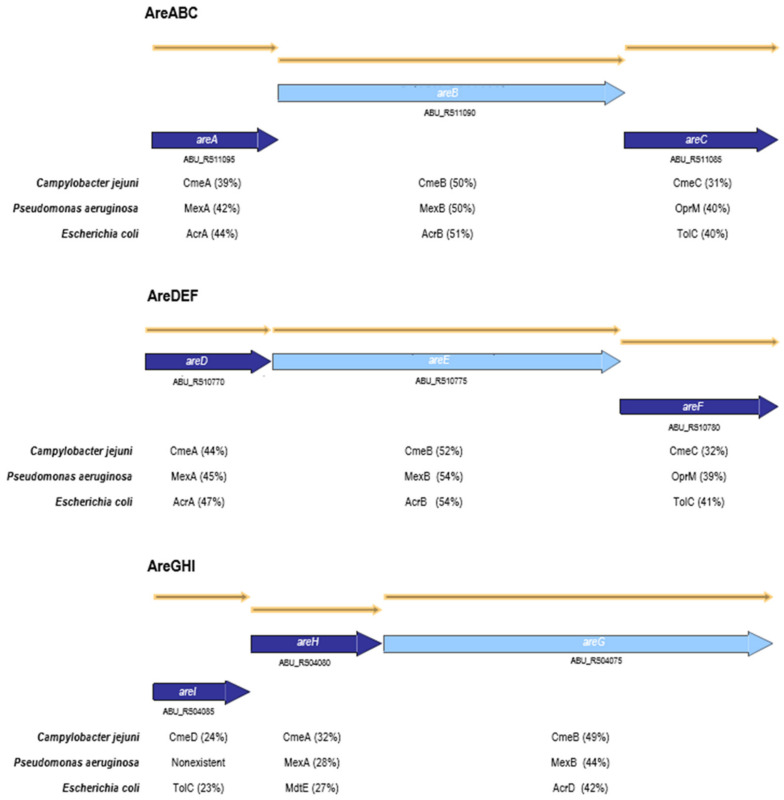
Genomic organization of AreABC, AreDEF and AreGHI efflux pumps. Open reading frames and their transcription directions are indicated by boxed arrows, and locus tag from *Aliarcobacter butzleri* RM4018 is identified below the arrow. Putative genes encoding the inner membrane protein of the efflux pump are marked in light blue. Amino acid identities (percentage of similarity) between protein homologs in *C. jejuni, P. aeruginosa* and *E. coli* are indicated for each homolog gene and were retrieved using BLASTP analysis.

AreABC, AreDEF and AreGHI are efflux pumps belonging to the RND family that were found encoded in the genome of *A. butzleri* (namely in the strain Ab_2811) [13]. A BLASTP analysis showed that the efflux pumps under study have homology with other RND efflux systems, namely from *C. jejuni*, *P. aeruginosa* and *E. coli*. The three structural components of these RND efflux systems are organized in a single operon containing outer membrane protein genes (Figure 1), similarly to the systems CmeABC and CmeDEF from *C. jejuni* or MexAB-OprM and MexEF-OprN from *P. aeruginosa*. This contrasts with other Gram-negative species, for which the RND efflux transporters operons lack genes coding for outer membrane proteins, which in turn may be located elsewhere in the chromosome, such as in the case of the AcrAB-TolC system from *Escherichia coli* [15,26,27,28].

Each component of these tripartite efflux pumps is necessary for the efflux, and the absence of one of these components turns the complex nonfunctional [26]. Thus, to evaluate the potential role of three RND-type efflux pumps in *A. butzleri*, a strain with a multidrug-resistant profile was selected, and three mutants of the inner membrane protein genes, *areB*, *areE* and *areG*, were generated by natural transformation. The three genes were interrupted with a kanamycin resistance marker, by insertional mutation, originating the following mutants: Ab_2811∆*areB*, Ab_2811∆*areE* and Ab_2811∆*areG*. The insertion of the resistance cassette was verified by polymerase chain reaction (PCR) (Figure S1), while the inactivation of gene expression was confirmed by reverse transcription (RT) PCR (Figure S2).

The contribution of the efflux pumps was evaluated by MIC determination of several classes of antibiotics, bile salts and biocides for the parental and the mutant strains. This evaluation makes it possible to ascertain if the inactivation of the efflux systems AreABC, AreDEF and AreGHI has an influence on the resistance of the strain and to infer about potential substrates of these pumps.

All mutant strains were more susceptible than the parental strain, but different susceptibility profiles were observed for the different mutants (Table 1). The differences in MIC values obtained for the 22 antimicrobials tested suggests that the substrates of the AreABC efflux pump are cephalexin, cefotaxime, erythromycin, clarithromycin, streptomycin, ciprofloxacin and moxifloxacin, for which considerable fold-changes were observed (between 4- and 32-fold decrease in the MIC) for the mutant Ab_2811∆*areB*. Furthermore, levofloxacin, doxycycline and acriflavine may also be potential substrates of AreABC system, despite only a 2-fold decrease in the MIC being observed. The role of AreABC in *A. butzleri* antibiotic resistance was previously described and evidenced for low to intermediate levels of erythromycin resistance [14].

Considering the AreDEF efflux pump, the impact of *areE* inactivation was less evident, with a decrease of only 4-fold for cephalexin and 2-fold for cefotaxime, erythromycin, clarithromycin, streptomycin, doxycycline and sodium cholate.

Regarding the AreGHI mutant, the results suggest that cephalexin, cefotaxime, amoxicillin, streptomycin, levofloxacin, tetracycline, doxycycline, chloramphenicol and bile salts are substrates of this efflux pump. In addition, a 2-fold decrease in the MIC for ampicillin, ciprofloxacin, sodium cholate, benzalkonium chloride and acriflavine was proved, when compared to the parental strain, suggesting that these compounds may also stand as possible substrates for this pump.

Overall, the three efflux pumps recognize a broad range of substrates, with all the efflux systems demonstrating to have a role in the extrusion of cephalexin, cefotaxime, streptomycin and doxycycline.

These results are in accordance with several studies carried out in RND efflux systems from different bacterial species, where the inactivation of genes encoding for efflux pump subunits results in increased susceptibility to different antimicrobials [15,16,27]. For example, CmeABC from *Campylobacter* spp. was described as being involved in the efflux transport of several classes of antibiotics, ethidium bromide and bile salts, proving that this system recognizes a broad range of structurally different substrates [28]. In the case of CmeDEF efflux system of *C. jejuni*, when the *cmeF* gene was interrupted, only a small decrease in MIC for ampicillin and cefotaxime was observed [29]. Therefore, the role of the CmeABC in *C. jejuni* resistance has been considered more relevant than the role of CmeDEF, with the first significantly influencing the intrinsic and acquired resistance of *Campylobacter* to various antimicrobials, such as macrolides and fluoroquinolones [28,30,31].

Accordingly, in our study, AreABC and AreGHI efflux systems demonstrated a greater influence in the extrusion of toxic compounds out of the cell than AreDEF, recognizing a different wide range of substrates. These results corroborate previous studies regarding the role of AreABC in *A. butzleri* resistance [14] and support, for the first time, the contribution of AreGHI as well. Besides that, AreDEF efflux pump has shown a selectivity for a few substrates; however, it does not seem to play such a significant role in the multidrug resistance of the *A. butzleri* strain Ab_2811.

**Figure 2:**
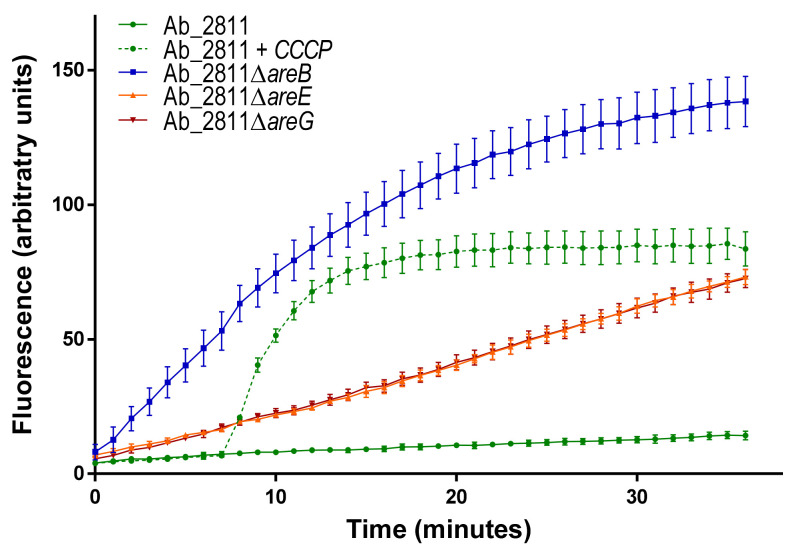
Accumulation of ethidium bromide after 37 min of incubation for parental *Aliarcobacter butzleri* Ab_2811 strain and derived mutants, in the absence (for mutants) or presence (for parental strain) of carbonyl cyanide m-chlorophenyl hydrazone at 50 µM. Data match to mean ± standard error of the mean (SEM) from three independent experiments.

To evaluate if the increase in susceptibility observed for mutant strains was a consequence of efflux loss, the intracellular accumulation of ethidium bromide (EtBr) was assessed for parental and mutant strains (Figure 2). A time-dependent increase in fluorescence was observed for all strains, with the Ab_2811∆*areB* mutant accumulating more EtBr than the parental strain and the other two mutants. When accumulation of ethidium bromide was assessed, fluorescence increased by ~5- to 9-fold after 37 min for mutant versus parental strain. Considering that a defective efflux results in dye accumulation and consequently an increase in fluorescence, these results support the functionality of the three efflux pumps of *A. butzleri* [32]. When the efflux pump inhibitor carbonyl cyanide m-chlorophenyl hydrazone was added to the parental strain, a ~6-fold increase was observed, confirming the contribution of efflux systems to the ethidium bromide accumulation, as previously described for *C. jejuni* [28].

### 2.2. Influence of Efflux System Impairment in Bacterial Growth and Relative Fitness

**Figure 3:**
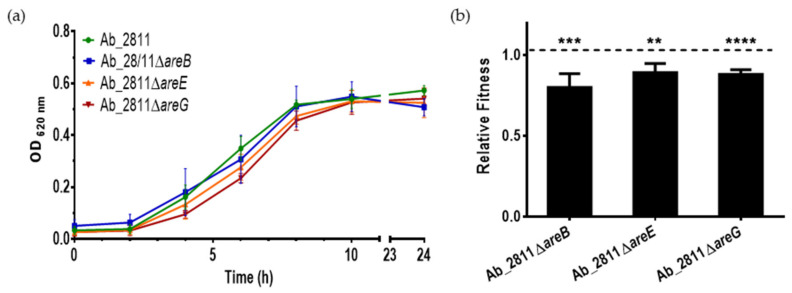
(**a**) Growth curves and (**b**) relative fitness of *Aliarcobacter butzleri* parental Ab_2811 strain and derived mutants. Data match to mean ± standard deviation (SD) from at least three independent experiments. ** *p* < 0.01; *** *p* < 0.001; **** *p* < 0.0001.

Several studies showed that the deletion of a gene from an efflux pump can affect bacterial growth and influence bacterial fitness [16,33,34]. To assess the effects, growth curves of the parental *A. butzleri* Ab_2811 strain and their mutants were constructed (Figure 3a). The mutant strains exhibited similar growth curves when compared with the parental strain. Despite the observed slight reduction in growth during the exponential phase, the mutations did not confer significant growth deficiency. This behavior is consistent with other studies showing that a mutation in the gene encoding the carrier protein of the RND-type efflux pump did not impair growth [23,24,35].

A fitness assessment was conducted for parental and mutant strains to determine if the inactivation of these genes could confer an advantage or disadvantage to the strain [36]. The pairwise competition assay showed that all three mutant strains underwent a fitness cost (Figure 3b), following the same trend observed by Pérez et al. (2012), where the disruption of the efflux system led to a reduction in bacterial fitness [23]. Overall, our results indicate that the efflux pumps under study participate in maintaining the biological competitiveness of *A. butzleri.* Indeed, it has been described that a variation of the expression of efflux pumps may induce specific changes in bacterial regulatory networks [16].

### 2.3. Effect of Inactivation of the RND Efflux Pumps in the Susceptibility to Oxidative and Bile Stress

In addition to conferring resistance to certain antibiotics, efflux pumps may have a role in resistance to oxidative stress. Oxidative stress occurs in bacteria, and in order to prevent the damage caused by the reactive oxygen species, a detoxification process occurs that involves the transport of these compounds by efflux pumps. RND efflux pumps have been associated with the extrusion of a variety of oxidative stress-inducing agents such as hydrogen peroxide and paraquat [37].

**Figure 4:**
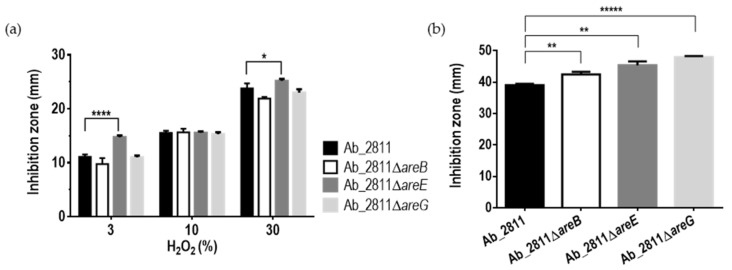
Oxidative stress susceptibility of *Aliarcobacter butzleri* parental Ab_2811 strain and derivate mutants, induced by (**a**) hydrogen peroxide and (**b**) paraquat. Data correspond to the mean ± SD of at least three independent experiments. * *p* < 0.05; ** *p* < 0.01; **** *p* < 0.0001; ***** *p* < 0.00001.

To validate this hypothesis, we tested the resistance to hydrogen peroxide and paraquat. Only the mutant Ab_2811Δ*areE* exhibited an increase in the inhibition diameter zone against 3% and 30% hydrogen peroxide when compared to the parental strain (Figure 4a). In contrast, all mutant strains showed an increased susceptibility to paraquat (Figure 4b), suggesting that these efflux pumps contribute to the bacterial tolerance to oxidative stress induced by paraquat.

These results are in accordance with a previous study showing that deletion of *cmeG* gene of *C. jejuni* resulted in increased susceptibility to hydrogen peroxide. The mechanism contributing to resistance to oxidative stress in *Campylobacter* is not yet known, but it has been hypothesized that the extrusion of toxic metabolites contributes to the detoxification process and therefore to oxidative stress resistance [37]. Furthermore, in *Salmonella enterica* serovar Typhimurium, the overexpression of the RND efflux pump MdsABC resulted in an increase in the resistance to oxidative stress induced by paraquat, demonstrating a role of efflux pumps in the defense mechanism against this stress [38].

Based on our results, we suggest that the AreDEF system may present a similar function to *cmeG* in *C. jejuni*, while the three systems may alleviate stress induced by paraquat, possibly playing a critical role in the oxidative balance necessary for normal physiology.

Bacteria that infect the gastrointestinal tract are exposed to several barriers, such as bile salts [39], which can disrupt cell membranes [40]. Efflux pumps may play a role in the bacterial resistance against these compounds. As an example, *C. jejuni* uses the CmeABC efflux pump as a mechanism for resistance to bile [21].

**Figure 5:**
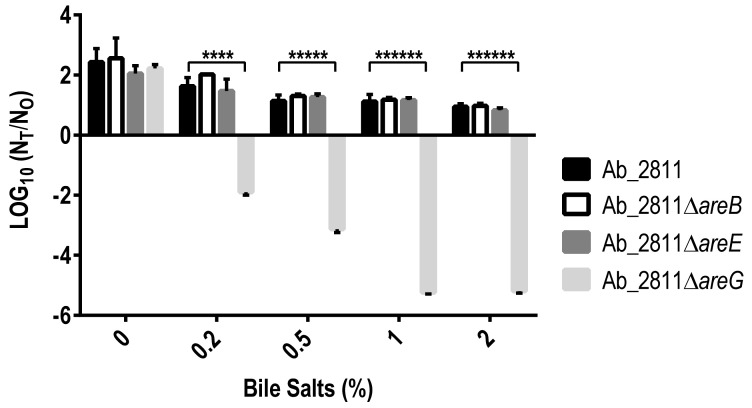
Bile stress susceptibility of *Aliarcobacter butzleri* parental Ab_2811 strain and derivate mutants. Data correspond to the mean ± SD of three independent experiments. **** *p* < 0.0001; ***** *p* < 0.00001; ****** *p* < 0.000001; The survival was determined as logarithm of N_T_/N_0_, where N_T_ corresponds to the colony-forming units (CFU)/mL after 6 h of incubation and N_0_ corresponds to CFU/mL at time 0.

Since *A. butzleri* is considered a pathogen capable of infecting the gastrointestinal tract [5] and the inactivation of *areG* gene led to a reduction in the MIC for bile salts, further studies were undertaken. For that, the parental and mutant strains were exposed to a physiological range of bile salt concentrations [41]. Overall, increasing concentrations of bile salts in the medium resulted in a decrease in the strains’ growth, while for the Ab_2811∆*areG* mutant, cellular death was observed for all the tested concentrations (Figure 5). Several studies have shown that efflux systems are relevant for bacterial survival in the gastrointestinal tract and that this may be their ancestral function [19,27]. In *C. jejuni*, it was demonstrated that the inactivation of the CmeABC efflux pump led to a decreased resistance of the bacterium to bile salts [21,28,29,42]. The role of the efflux systems in the bacterium’s survival in the presence of bile salts has also been described for other bacteria associated with human intestinal tract infections, such as *Vibrio cholerae* and *Bacteroides fragilis* [16,43]. Accordingly, our results strongly support a role of the AreGHI system in *A. butzleri* survival in the gastrointestinal tract, likely influencing the capacity of *A. butzleri* survival in response to this defense mechanism of the host, while promoting bacterial adaptation to the intestinal tract.

### 2.4. Impact of the Inactivation of RND Efflux Pumps on Motility and Biofilm Formation Ability

**Figure 6:**
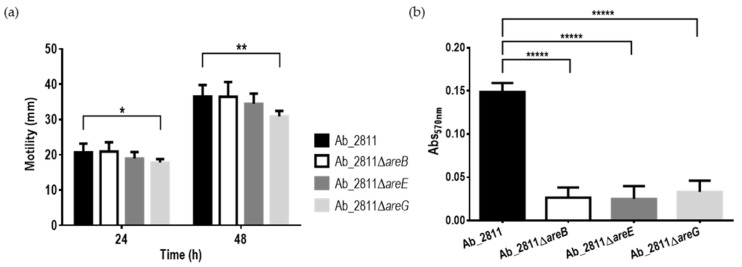
(**a**) Motility ability and (**b**) biofilm formation ability of *Aliarcobacter butzleri* parental Ab_2811 strain and derivate mutants. Data are the mean ± SEM of at least three independent experiments. * *p* < 0.05; ** *p* < 0.01; ***** *p* < 0.00001.

Motility and biofilm formation are two of the virulence mechanisms described for *A. butzleri* [5,6], being frequently associated with colonization and bacterial persistence, respectively [31,44]. A change in the expression of the efflux pumps can have an impact on the motility, which, in turn, may be associated with the formation of biofilms, influencing the virulence of the bacterium [45]. To assess whether these bacterial virulence characteristics were affected by inactivation of one of the subunits of the efflux pumps under study, motility and biofilm formation assays were performed for the parental and the mutant strains (Figure 6). Regarding motility, the migration diameter of all the mutant strains decreased; however, only the Ab_2811Δ*areG* mutant demonstrated a significant decrease when compared to the parental strain (Figure 6a). A decrease in motility has already been described for other bacteria, induced by the inactivation of an RND-type efflux pump [34,46].

The formation of biofilms was quantified by the crystal violet colorimetric assay. The inactivation of the three efflux systems led to a significant decrease in biofilm formation when compared to the parental strain (Figure 6b). Several studies described a similar behavior, where a deletion or inhibition of efflux pumps was associated with a decrease in biofilm formation [47,48]. For example, when the *adeB* gene of the AdeABC efflux system from *A. baumannii* was deleted, the mutant had a significant defect in biofilm formation. Moreover, mutants of *Salmonella* Typhimurium lacking *tolC* and *acrB* genes had compromised biofilm formation ability [49,50]. RND efflux pumps also have the ability to extrude quorum-sensing signals, with an impact on the process of intercellular communication, which in turn is associated with biofilm formation [16].

### 2.5. Impact of the Inactivation of RND Efflux Pumps on Human Serum Susceptibility

**Figure 7:**
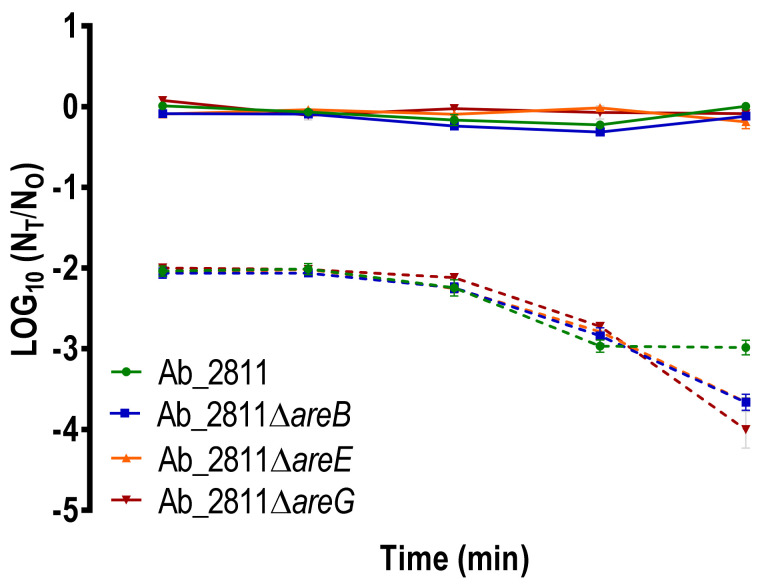
Susceptibility of *Aliarcobacter butzleri* parental Ab_2811 strain and derivate mutants to human serum. Death of the bacteria on PBS (solid lines) and serum (dash lines). Data correspond to the means ± SD of three independent experiments.

To verify the serum’s bactericidal effects on parental and mutant strains, we performed the serum killing assay. PBS was used as control of the experiment, and no killing effects were observed for all the strains. Overall, the survival of the mutant strains in healthy human serum was shown to be significantly different from that observed for the parental strain (Figure 7). Indeed, after 180 min of exposure, all the mutants demonstrated a significant increase in their susceptibility to human serum (*p* < 0.01). These results are in agreement with a study performed in *Stenotrophomonas maltophilia*, where the deletion of the RND efflux pump SmeYZ resulted in increased susceptibility to human serum [51].

### 2.6. In Vitro Adhesion to and Invasion into Caco-2 Cells

**Figure 8:**
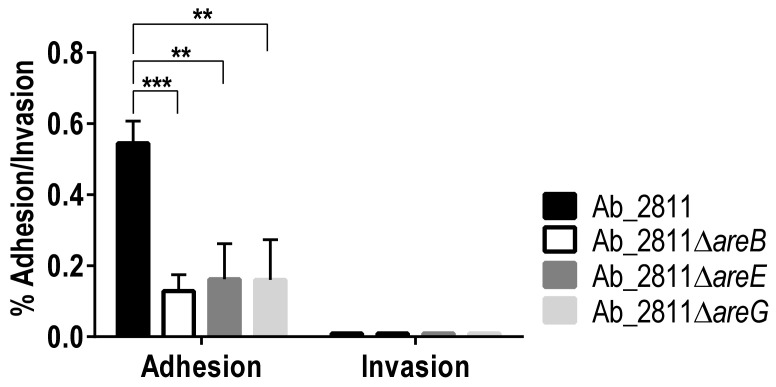
Adhesion and invasion assays for parental *Aliarcobacter butzleri* Ab_2811 strain and derivate mutants. Data correspond to the mean ± SEM of three independent experiments. ** *p* < 0.01; *** *p* < 0.001.

RND-type efflux pumps are recognized for their role in resistance as well as in virulence [52]. Since capacities for adhesion to and invasion into host cells are required for successful colonization and infection [53], these in vitro assays were performed to evaluate virulence for parental and efflux pumps mutant strains. Compared to the parental strain, all mutants exhibited a significant decrease in the capacity for adhesion to Caco-2 cells (Figure 8). However, no difference was observed regarding invasion ability between the parental and mutant strains, probably due to the very low percentage of invasion observed for all the strains (Figure 8).

Our results are in accordance with a previous study carried out in *Salmonella enterica*, where the lack of AcrB led to a decrease in adhesion and invasion. In that study, the reduction in virulence observed for the mutant strains was caused by the loss of the efflux function [24]. Supporting this, several studies have shown that RND efflux pumps play a relevant role in the virulence of bacteria. For example, in *C. jejuni*, the CmeABC system is needed for gut colonization of 1-year-old chicks [21]; in *Moraxella catarrhalis*, a deletion of the AcrAB-oprM system led to a decrease in invasion of the mutant into pharyngeal epithelial cells [54]; and in *Vibrio cholerae*, RND efflux systems are essential for the colonization of the small intestine in mice [55]. Overall, all the *A. butzleri* efflux systems under study showed a potential role in adhesion ability, thus contributing to bacterial virulence and infection of host cells.

In summary, the results suggest that the *A. butzleri* efflux systems AreABC, AreDEF and AreGHI have a role in the extrusion of several antimicrobial agents, such as antibiotics, bile salts and biocides. In addition, results showed that these pumps are also relevant elements in the defense against oxidative stress, while AreGHI has a role in the survival of the bacterium in presence of bile salts. Finally, all the efflux systems tested showed a role in the ability to form biofilms, the ability to survive in human serum and the ability to adhere to intestinal cells. Thus, overall, the three efflux pumps studied have been shown to contribute to antimicrobial resistance and also to virulence of *A. butzleri*. Our results are in agreement with the well-known role of RND efflux systems in antibiotic resistance, while their role in other relevant physiological processes, namely those associated with bacterial virulence, has also been described for several pathogens, such as *C. jejuni*, *S. maltophilia* and *P. aeruginosa* [16]. This study produced relevant data towards a better understanding of the emerging pathogen *A. butzleri*, while allowing the identification of putative targets for new therapeutic strategies to fight this infection.

## 3. Materials and Methods

### 3.1. Bacterial Strains and Growth Conditions

The *A. butzleri* strain Ab_2811 was isolated from a poultry carcass neck skin [56] and was used as the parental strain for this study. Bacterial cells were stored at −80 °C in brain heart infusion (BHI) containing 20% glycerol and were routinely cultured on blood agar base (BA, Oxoid, Basingstoke, England) supplemented with 5% (*v*/*v*) of defibrinated horse blood or in tryptic soy agar (TSA, VWR, Leuven, Belgium) medium and incubated at 30 °C for 24 h, in aerobic conditions. For growth in broth culture, the *A. butzleri* strains were transferred to 10 mL of tryptic soy broth (TSB) with an initial optical density at 620 nm (OD_620_ nm) of 0.02 and incubated overnight at 30 °C under aerobic conditions.

### 3.2. Construction of Aliarcobacter butzleri Efflux Pump Mutants

The construction of the *A. butzleri* Ab_2811 mutants was performed using insertional mutagenesis, where the *areB*, *areE* and *areG* genes, encoding for the inner membrane protein of each of the three RND efflux systems under study, were interrupted by a kanamycin resistance cassette (*aphA-3*). The *aphA-3* cassette was obtained by enzymatic digestion of pUC18-K2 plasmid with BamHI and KpnI, followed by purification. Then, amplification of the upstream and downstream regions of each gene was performed using the primers presented in Table S1. Primers were designed with a tail sequence that allows hybridization with upstream and downstream regions of the kanamycin resistance marker. The overlap-extension PCR was used to construct the transformant DNA fragment. This fragment consisted of an *aphA-3* cassette flanked by the amplified fragments of an upstream and a downstream region of the genes under study. After purification, the transformant DNA fragment was used for the natural transformation of *A. butzleri* through a biphasic method based on a protocol previously described, with some modifications [57]. Briefly, *A. butzleri* Ab_2811 was cultured in BA at 30 °C for 24 h in a microaerophilic atmosphere (6% O_2_, +/− 7.1% CO_2_ and 3.6% H_2_). Then, 5 × 10^9^ CFU were resuspended in TSB and transferred to a test tube containing 1.5 mL of BA and incubated under the same conditions for 6 h. At this point, 2 μg of the transformant DNA was added and tubes were further incubated for 5 h. After incubation, the suspension was transferred to a BA plate supplemented with 50 μg/mL of kanamycin and incubated at 30 °C under microaerophilic atmosphere, for 5 days. The transformation was confirmed by PCR through analysis of the fragment size, and the gene expression was evaluated by RT-PCR technique (Table S1).

### 3.3. Antimicrobial Susceptibility Test

The minimum inhibitory concentration (MIC) was determined by broth microdilution method [58] for several classes of antibiotics, including β-lactams, quinolones, aminoglycosides, tetracyclines, macrolides and phenicols, and for different bile salts, disinfectants and a germicide. The MIC of compounds tested was assessed followed by spectrophotometric confirmation at 620 nm, using the value of 0.05 as a cut-off of the optical density. This test was performed in duplicate and in at least three independent assays.

### 3.4. Ethidium Bromide Accumulation Assay

The EtBr accumulation in parental and mutant *A. butzleri* strains was evaluated as previously described, with minor modifications [14,28]. Cultures were grown until mid-exponential phase, collected by centrifugation, washed with phosphate-buffered saline (PBS) and resuspended at OD_620_ nm of 0.2. The suspension was transferred to a black 96-well plate with a clear bottom (Greiner Bio-One, Frickenhausen, Germany). After incubation for 10 min at 30 °C, EtBr was added to each well to a final concentration of 2 µg/mL. Fluorescence was recorded with a Spectramax Gemini XS spectrofluorometer (Molecular Devices LLC, San Jose, CA, USA) at excitation and emission wavelengths of 530 and 600 nm, respectively. For the accumulation in the presence of efflux pump inhibitor, after 7 min of readings, carbonyl cyanide m-chlorophenyl hydrazone was added to each well to Ab_2811 at a final concentration of 50 µM. Each assay was performed in triplicate and in three independent assays.

### 3.5. Growth Curves and Competition Experiments of the Parental and Mutant Strains

The growth curves of *A. butzleri* parental and mutant strains were determined as described previously [58], and competition experiments were performed using pairwise competition assay for parental and mutant strains [58]. Briefly, a fresh culture in 10 mL of TSB was started by adding equal volumes of a preculture of parental and mutant strains. This culture was incubated at 30 °C for 24 h under aerobic condition. Samples were taken at time 0 and 24 h of incubation and the total and mutant populations were counted by plating successive dilutions on TSA and TSA supplemented with 50 μg/mL of kanamycin, respectively. The relative fitness of each mutant was determined by the ratio of the Malthusian parameter of the mutant per the parental. Both assays were repeated at least three independent times.

### 3.6. Stress Susceptibility Assays

The oxidative stress tolerance was determined by the disk diffusion assays, where TSA was inoculated with a cellular suspension adjusted to OD_620_ nm of 0.2 using a cotton swab, and sterile paper disks were spotted with 5 μL of 3%, 10% and 30% hydrogen peroxide (H_2_O_2_) and 5 μL of 250 mM methyl viologen (paraquat). After incubation at 30 °C in aerobic atmosphere for 24 h, the zone of growth inhibition diameter was measured. To evaluate bile stress, an overnight culture of the parental and mutant strains was used to inoculate a culture in the absence and presence of bile salts, with a range from 0 to 2% (Sigma-Aldrich, St. Louis, MO, USA). At 0 and 6 h, the drop-plate method was used for determination of CFU/mL. These assays were performed three independent times.

### 3.7. Motility Assay

The motility of parental and mutant strains was evaluated as previously described [58]. The center of a plate of TSA medium with 0.4% of agar was stabbed with 5 μL of mid-exponential phase culture. Motility was measured at 24 and 48 h, after incubation at 30 °C in aerobic atmosphere. This assay was performed at least three independent times.

### 3.8. Biofilm Formation Ability

The ability of *A. butzleri* to form biofilm was evaluated by the protocol described by Reeser et al. (2007), with some modifications [59]. An overnight culture was diluted to an OD_620_ nm of 0.2, and 100 μL of the bacterial suspension was applied in nonuplicate on a round-bottom polystyrene 96-well microtiter plate. Wells with only medium were used as negative control. After 48 h of incubation at 30 °C in aerobic atmosphere, the medium was removed, and the plate was incubated for 1 h at 55 °C. Then, the biofilm was stained with 100 μL of crystal violet at 0.1% (*w*/*V*) in deionized water and incubated for 15 min at room temperature, followed by washing of the wells three times with distilled water and incubation again at 55 °C for 15 min. After drying, the biofilm was destained with 120 μL of a 30% methanol and 10% acetic acid solution. One hundred microliters was moved to a new microtiter plate and the absorbance at 570 nm was determined. The assay was performed at least three independent times.

### 3.9. Serum Killing Assays

Serum bactericidal assays were performed according to the protocol previously described by O’Shaughnessy et al. (2012), with some modifications [60]. Blood was collected from a healthy adult, and serum was separated by centrifugation at 2000 rpm for 10 min at 4 °C and frozen in aliquots at −80 °C in sterile cryogenic tubes. For the assay, the parental and mutant strains at mid-exponential phase were washed and resuspended in PBS at a final concentration of ~10^7^ CFU/mL with 90% of serum. As control, serum was replaced with PBS. The assay was performed at 30 °C, and viable cells were counted by successive dilution at the time points of 0, 15, 30, 45, 90 and 180 min. This assay was done three independent times.

### 3.10. Adhesion and Invasion Assays

Adhesion and invasion assays were performed in the Caco-2 human intestinal epithelial cell line. The Caco-2 cells were grown in tissue culture flasks in Dulbecco’s modified Eagle medium supplemented with 10% fetal bovine serum, 1% nonessential amino acids, 100 µg/mL streptomycin and 100 U/mL penicillin and incubated at 37 °C in 5% of CO_2_. For assays, 24-well plates were seeded with 1 × 10^5^ cells/ well and incubated for 48 h. To determine adhesion, monolayers were infected at an MOI of 100 and incubated for 2 h in the same atmospheric conditions. Cells were washed three times and lysed with 0.1% Triton X-100. For invasion of Caco-2 cells, a gentamicin protection assay was performed. After 2 h of infection, extracellular bacteria were killed by incubating with 125 µg/mL of gentamicin for 1 h. Then, cells were washed to remove residual antibiotic and lysed with 0.1% Triton X-100. Percentage of adherent and invading bacteria was determined by successive dilution of lysates and plating by drop-plate method. Each assay was performed at least three independent times, in triplicate.

## Figures and Tables

**Table 1:** Minimum inhibitory concentration of several classes of antibiotics, bile salts, disinfectants and germicides for the parental and *areB*, *areE* and *areG* mutant strains of *Aliarcobacter butzleri*.

Antimicrobial	MIC (μg/mL) [Fold Reduction]			
	Ab_2811	Ab_2811∆*areB*	Ab_2811∆*areE*	Ab_2811∆*areG*
β-Lactams				
Ampicillin	128	128 [ND]	128 [ND]	**64 [2]**
Cephalexin	256	**8** [32]	**64 [4]**	**0.0625 [4096]**
Cefotaxime	64	**2 [32]**	**32 [2]**	**0.0625 [1024]**
Amoxycillin	64	64 [ND]	64 [ND]	**16 [4]**
Macrolides				
Erythromycin	16	**2 [8]**	**8 [2]**	16 [ND]
Clarithromycin	16	**1 [16]**	**8 [2]**	16 [ND]
Aminoglycosides				
Gentamycin	1	1 [ND]	1 [ND]	1 [ND]
Kanamycin *	4	>512	>512	>512
Streptomycin	8	**0.5 [16]**	**4 [2]**	**2 [4]**
Quinolones				
Nalidixic acid	>256	>256 [ND]	>256 [ND]	>256 [ND]
Ciprofloxacin	32	**8 [4]**	32 [ND]	**16 [4]**
Levofloxacin	64	**32 [2]**	64 [ND]	**16 [4]**
Moxifloxacin	32	**8 [4]**	32 [ND]	32 [ND]
Tetracyclines				
Tetracyclines	8	8 [ND]	8 [ND]	**0.5 [16]**
Doxycycline	8	**4 [2]**	**4 [2]**	**1 [8]**
Phenicol				
Chloramphenicol	32	32 [ND]	32 [ND]	**8 [4]**
Bile salts				
Sodium cholate	8000	8000 [ND]	**4000 [2]**	**4000 [2]**
Sodium deoxycholate	32,000	32,000 [ND]	32,000 [ND]	32,000 [ND]
Bile salts mix	50,000	50,000 [ND]	50,000 [ND]	**6250 [8]**
Disinfectants				
Chlorhexidine	2	2 [ND]	2 [ND]	2 [ND]
Benzalkonium chloride	32	32 [ND]	32 [ND]	**16 [2]**
Germicide				
Acriflavine	32	**16 [2]**	32 [ND]	**16 [2]**

ND, no observed MIC difference. * A kanamycin resistance marker was used to inactivate *areB*, *areE* and *areG* genes. Changes of at least 2-fold are indicated in bold type.

## Data Availability

Data are contained within the text and the Supplementary Materials.

## Supplementary Materials

The following are available online at https://www.mdpi.com/article/10.3390/antibiotics10070823/s1, Table S1: Primers used in PCR in this study, Figure S1. Confirmation of constructed mutants by PCR, Figure S2. Confirmation of gene expression by RT-PCR.
