# Supplementary material for: RND Efflux Systems Contribute to Resistance and Virulence of Aliarcobacter butzleri
Source: Antibiotics (Basel). 2021 Jul 6;10(7):823. doi: 10.3390/antibiotics10070823 (PMC8300790; doi:10.3390/antibiotics10070823)
Supplement: Supplementary file 1 [file antibiotics-10-00823-s001.zip › antibiotics-1262027-supplementary.pdf]

antibiotics

MDPI

Table S1. Oligonucleotide sequences used in this study.

Identification of primers	Sequence
Primers for mutant construction	
areB_A1	5′-TTGAAATAAGGGCTACTTACTCAGG-3′
areB_A2	5′-TTATTCCTCCTAGTTAGTCACTCCAACACCATCTACTCTTGC-3′
areB_B1	5′-TACCTGGAGGGAATAATGATCCATTAGGAATTATTGGTGCAG-3′
areB_B2	5′-GTTCGCTCTGGCTTGCAAAT-3′
areE_A1	5′-TTCATCAACAGGAAATGCAAG-3′
areE_A2	5′-TTATTCCTCCTAGTTAGTCACAAATACGCATAGCATATTGACC-3′
areE_B1	5′-TACCTGGAGGGAATAATGAGCTGCACTTTATGAAAGTTGGTC-3′
areE_B2	5′-ATTTGCTCCTGCTCCTGTTG-3′
areG_A1	5′-AAACCCAGTTTTTGCTGGTG-3′
areG_A2	5′-TTATTCCTCCTAGTTAGTCAGCATCATCAGGATTTGTTCC-3′
areG_B1	5′-TACCTGGAGGAAATAATGAATCAGCTGGACAGGAACTGC-3′
areG_B2	5′-CTTCAAGTGCCGCATCAAC-3′
Primers for mutant confirmation	
areB_rtpcr_F	5′-TGGAAATGGACAAAATGGTG-3′
areB_rtpcr_R	5′-TCCAAGCCCTGGAATAGAAG-3′
areE_rtpcr_F	5′-TCAATTCGTTCTGATGATTCTGA-3′
areE_rtpcr_R	5′-TCTATAAGGAGCTTCACCTTGGA-3′
areG_rtpcr_F	5′-TGGCTATTACAGCAACAAATGAG-3′
areG_rtpcr_R	5′-TGAATTAATTGGTAAAAGCTCACC-3′

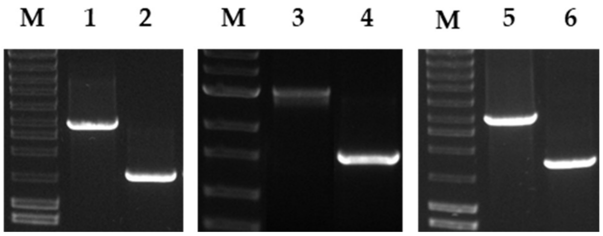

Figure S1. Confirmation of the mutants constructed by PCR. Lanes 1, 3 and 5: PCR products using specific primers for *areB*, *areE* and *areG* for the parental strain Ab_2811, respectively; 2, 5 and 6: PCR products using specific primers for *areB*, *areE* and *areG* for the mutants Ab_2811Δ*areB*, Ab_2811Δ*areE* and Ab_2811Δ*areG*, respectively; M: molecular weight.

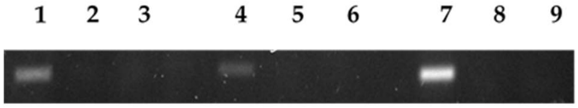

Figure S2. Expression of the mutated gene in each strain was determined by RT-PCR. Lanes 1, 4 and 7: RT-PCR products using specific primers for areB, areE and areG for the parental strain Ab_2811, respectively; 2, 5 and 8: RT-PCR products using specific primers for areB, areE and areG for the mutants Ab_2811Δ*areB*, Ab_2811Δ*areE* and Ab_2811Δ*areG*, respectively; 3, 6 and 9: negative controls.
